# ﻿A new species of *Sarsinebalia* (Crustacea, Leptostraca) from Japan

**DOI:** 10.3897/zookeys.1097.74243

**Published:** 2022-04-26

**Authors:** Takuma Hirata, Tomohiko Kikuchi

**Affiliations:** 1 National Institute for Environmental Studies, Biodiversity Division, Ecological Risk Assessment and Control Section, Onogawa, Tsukuba, Ibaraki 305-8506, Japan National Institute for Environmental Studies, Biodiversity Division Tsukuba Japan; 2 Graduate School of Environment and Information Sciences, Yokohama National University, 79-2 Tokiwadai, Hodogaya, Yokohama, Kanagawa 240-8501, Japan Yokohama National University Yokohama Japan

**Keywords:** Ago Bay, biodiversity, Malacostraca, taxonomic key

## Abstract

A new species of Leptostraca, *Sarsinebaliaagoensis***sp. nov.**, from Ago Bay, Japan is described from specimens found at a depth of 120 m. The new species differs from other known *Sarsinebalia* species as follows: the compound eye has three distal lobes; the anterior margin of the first antennal segment has one distal process covered with setae; and the lateral margin of pleopod 1 exopod bears 5–6 simple, robust spines. A taxonomic key to all species of *Sarsinebalia* is also provided.

## ﻿Introduction

The genus *Sarsinebalia* Dahl, 1985 belongs to the family Nebaliidae (Leptostraca: Nebaliacea). The type species of the genus, *Sarsinebaliatyphlops* (Sars, 1870), was initially included in the genus *Nebalia* Leach, 1814. However, in the revision of the European shelf species by Dahl (1985) it was pointed out that *Nebaliatyphlops* showed several morphological characters not present in any known *Nebalia* species at the time, i.e., rostrum with a ventral keel and terminal spine; compound eye disc-shaped and lacking pigment or externally discernible visual elements; first pleopod exopod lacking a row of serrated spines on the lateral border; and second maxilla exopod shorter than first article of endopod. Therefore, Dahl established the genus *Sarsinebalia* based on these characters. However, the validity of the genus *Sarsinebalia* was challenged by the phylogenetic analyses of Walker-Smith and Poore (2001), and later reconsidered by Moreira et al. (2003, 2021) and McCormack et al. (2016). The genus *Sarsinebalia* is currently composed of seven species that have been recorded from the Bay of Biscay (Ledoyer 1998), the sea near New Caledonia (Ledoyer 2000), the Atlantic coast of the Iberian Peninsula (Moreira et al. 2003; Sampaio et al. 2016), the Kuril-Kamchatka Trench (Petryashov 2016), the British Isles (McCormack et al. 2016), French Atlantic and Mediterranean waters (Latry and Droual 2020), and the Gulf of Cadiz (Moreira et al. 2021).

Three leptostracan species had long been reported from waters off the coast of Japan: *Nebaliabipes* (Fabricius, 1780), *Paranebalialongipes* (Willemoes-Suhm, 1875), and *Nebaliopsistypica* (Sars, 1887). Later, Hirata et. al. (2019) described *Nebaliatagiri* Hirata, Fujiwara & Kikuchi, 2019 from a hydrothermal field in Kagoshima Bay, Japan. Here, we report three specimens belonging to the genus *Sarsinebalia* collected from a depth of 120 m near Ago Bay, Japan in 1986. These specimens represent a new species that is described here as *Sarsinebaliaagoensis* sp. nov.. There have been few studies of Leptostraca in waters near Japan, and previous findings have not explained the full diversity. This report is considered to be a very important discovery for the evaluation of the diversity of Leptostraca in waters near Japan.

## ﻿Materials and methods

Samples were collected by ORI dredge; specimens ware sorted in the laboratory, and then identified and sexed. The following measurements were considered: Total length (**TL**: measured from the articulation between the rostrum and the carapace to the posterior end of the caudal furca), lateral carapace length (**LCL**: measured from the anterodorsal margin to the posteromedian margin of the carapace), carapace height (**CH**: measured between the dorsal and ventral margins), and rostrum length (**RL**: measured along the midline). Drawings were made with the aid of a camera lucida on a stereomicroscope (Model SMZ-10; Nikon Corporation, Japan). The type material of the new species is deposited at the National Museum of Nature and Science, Tokyo (**NSMT**).

## ﻿Taxonomy

### ﻿Order Leptostraca Claus, 1880

**Suborder Nebaliacea Calman, 1904**

**Family Nebaliidae Samouelle, 1819**

#### Genus *Sarsinebalia* Dahl, 1985

##### ﻿ Sarsinebalia agoensis sp. nov.

Taxon classificationAnimaliaLeptostracaNebaliidae

43C329B5-0F8A-5B35-9300-33BB514055CE

http://zoobank.org/04D34FE7-C3B2-42C0-AD9D-B5E1DEA2DF7E

**Figure 1.**
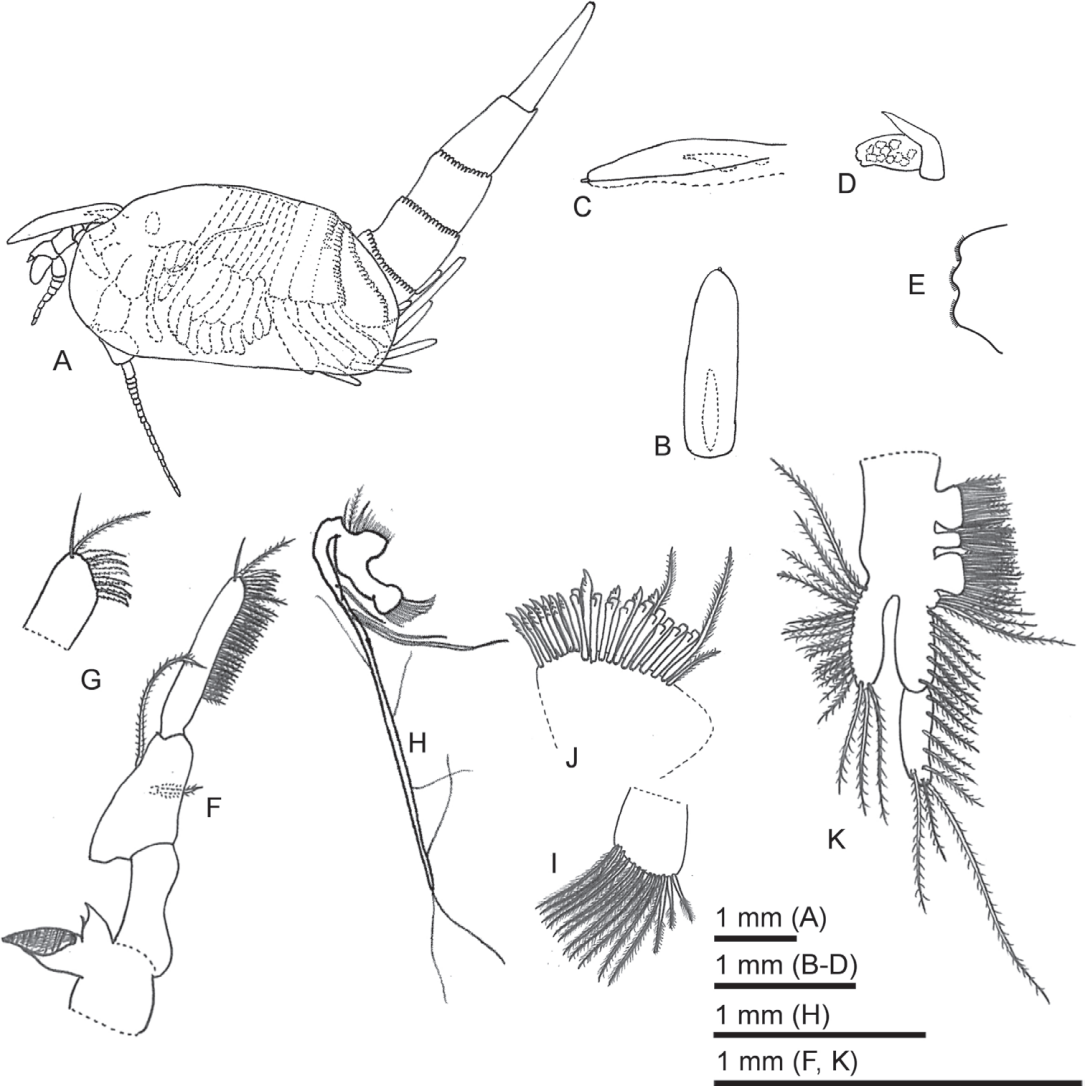
*Sarsinebaliaagoensis* sp. nov. **A** female holotype, lateral view **B** rostrum, dorsal view **C** rostrum, lateral view **D** compound eye **E** detail of eye lobes **F** mandible **G** detail of mandibular palp article 3, distal end **H** first maxilla **I** detail of first maxilla proximal endite **J** detail of first maxilla distal endite **K** second maxilla.

**Figure 2.**
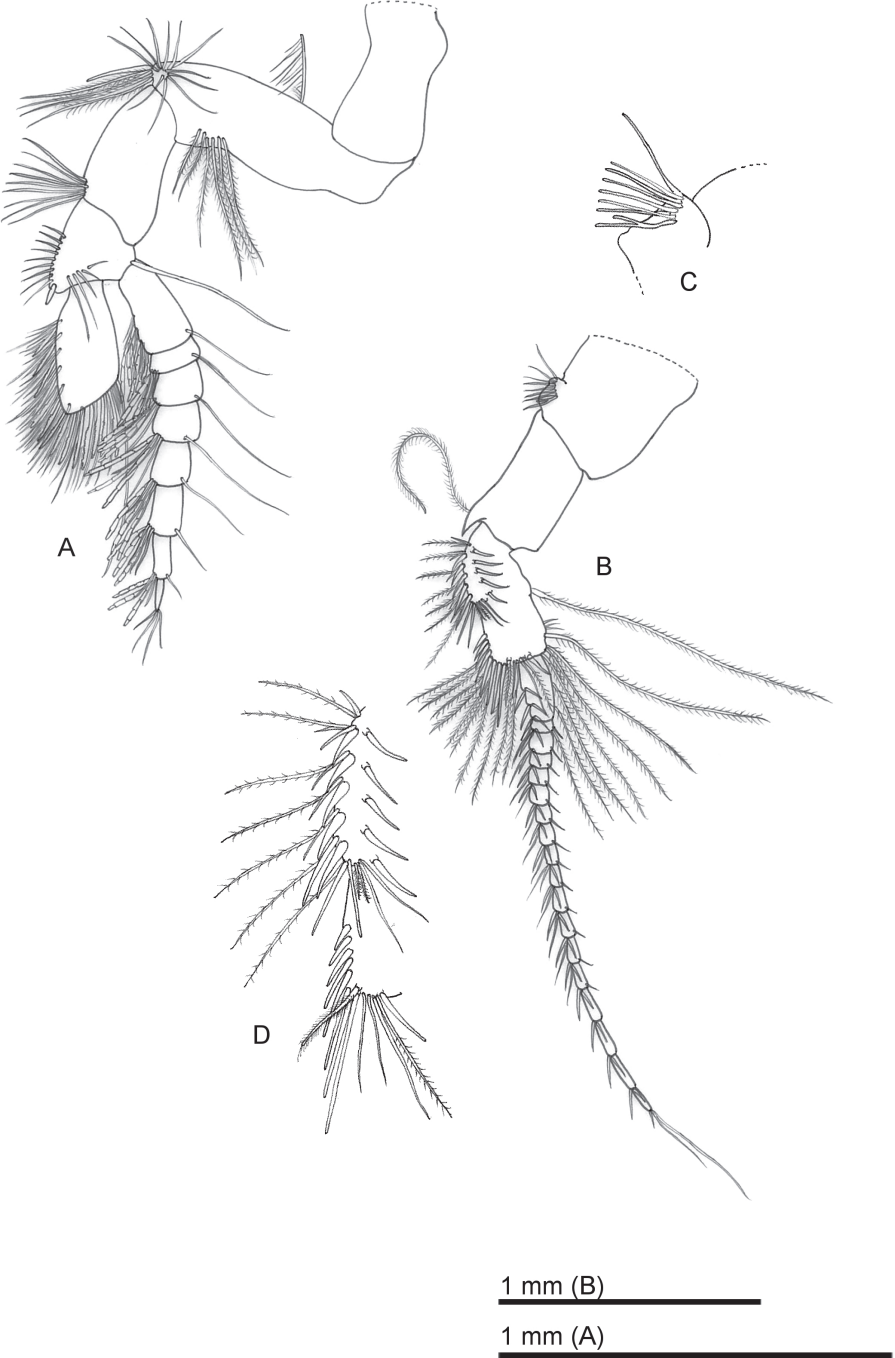
*Sarsinebaliaagoensis* sp. nov. **A** antennule **B** antenna **C** detail of process on antenna peduncle article 1 **D** detail of row of spines and setae on antenna peduncle article 3.

**Figure 3.**
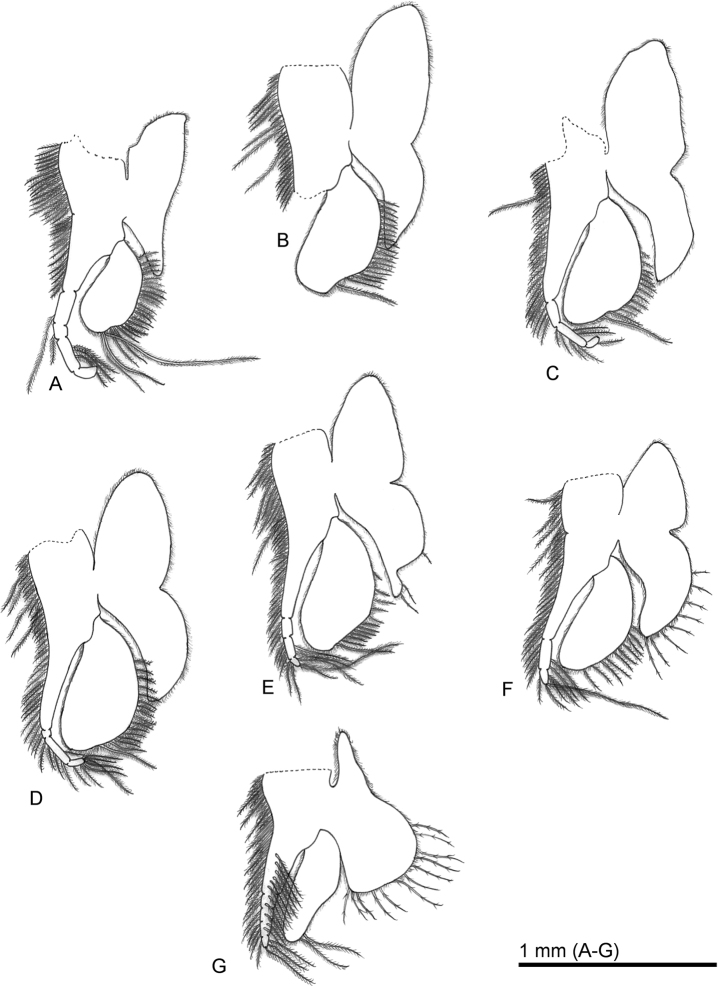
*Sarsinebaliaagoensis* sp. nov. **A** thoracopod 1 **B** thoracopod 3 **C** thoracopod 4 **D** thoracopod 5 **E** thoracopod 6 **F** thoracopod 7 **G** thoracopod 8.

**Figure 4.**
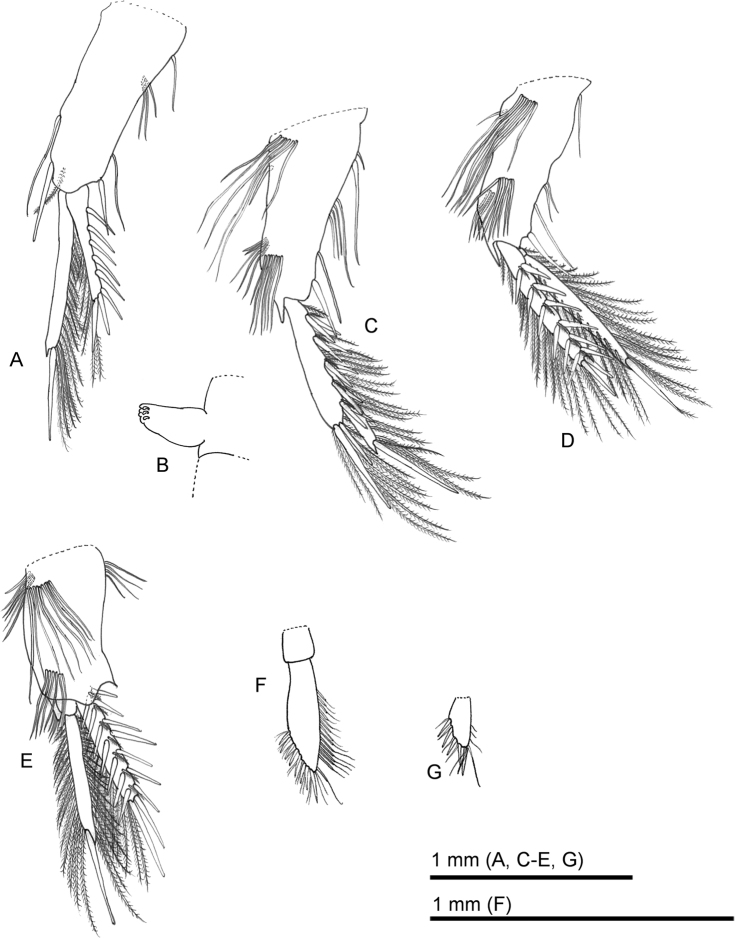
*Sarsinebaliaagoensis* sp. nov. **A** pleopod 1, lateral view **B** detail of pleopod 1 appendix interna **C** pleopod 2, lateral view **D** pleopod 3, lateral view **E** pleopod 4, lateral view **F** pleopod 5, ventral view **G** pleopod 6, ventral view.

**Figure 5.**
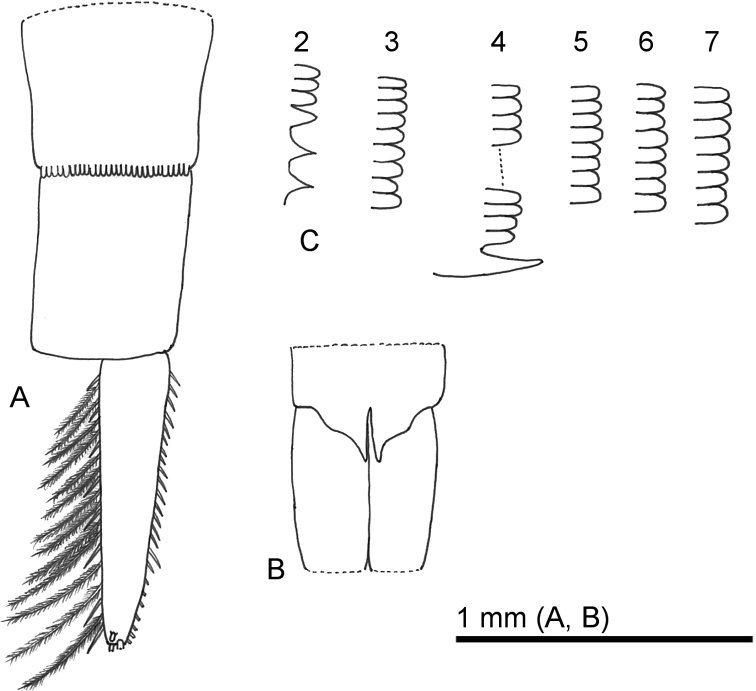
*Sarsinebaliaagoensis* sp. nov. **A** telson and right furcal ramus, dorsal view (left ramus not illustrated) **B** anal plates, ventral view **C** detail of denticles of pleonites 2–7 posterior margin.

Figs 1 , 2 , 3 , 4 , 5

###### Diagnosis.

Carapace reaching pleonite 5. Rostrum long and narrow, about 3.8 times as long as wide, bearing a thin terminal spine. Compound eyes subrectangular, with three lobes on terminal margin. Article 4 of antennule with a row of nine simple setae and one robust distal spine. Antennular scale about twice as long as wide. Article 1 of antenna with one rounded process on anterior margin, covered by setae. Article 1 of endopod of second maxilla sub-equal in length to article 2, exopod not beyond article 1 of endopod. Article 2 of mandibular palp with one long plumose and one thin plumose seta. Pleonites 2–7 with distally rounded denticles along posterior border. Epipod of thoracopod 8 distinctly broader than thoracopods 1–7. Exopod of pleopod 1 with a single row of simple spines along lateral margin. Anal plate with lateral “shoulder”. Furcal rami shorter than combined length of pleonite 7 and telson.

###### Examined material.

Three ♀♀ (TL: 4.5–8.0 mm) collected from the Japanese Pacific coast near Ago Bay, during the R/V “Tansei Maru” KT 86-6 cruise (Atmosphere and Ocean Research Institute, The University of Tokyo); 34°12'00N, 136°43'00E; 120 m; May 1986.

###### Types material.

***Holotype***: (NSMT-Cr 28987), adult ♀, 8.0 mm **TL. *Paratypes***: two adult ♀♀ (**TL**: 5.6–6.5 mm) (NSMT-Cr 28988, NSMT-Cr 28989).

###### Description.

**Female holotype**:

***Carapace*** (Fig. 1A): Oval, reaching lateral side of pleonite 5. LCL about 4.2 mm; carapace ca. twice longer than high.

***Rostrum*** (Fig. 1B, C): Long and narrow, ventral keel long and narrow, with thin terminal spine. RL about 1.6 mm; approximately 3.8 times as long as wide.

***Compound eye*** (Fig. 1D, E): Subrectangular in shape, ommatidial part covering most of eyestalk, with three serrated lobes on terminal margin (Fig. 1E). Supraocular plate extending to about half of eyestalk.

***Antennule*** (Fig. 2A): Peduncle composed of four articles. Article 1 about twice as long as wide. Article 2 longer than article 3, with a single plumose seta on anterior margin, four long and two short plumose setae arising subterminally, one spine-like seta, four plumose setae, and a cluster of simple setae on anterior margin, respectively. Article 3 expanded distally, with a terminal cluster of simple setae on anterior margin. Article 4 about half as long as article 3, with a row of nine simple setae and one spine distally, a row of five simple setae on anterior face, and a long robust seta on posterior margin. Antennular scale oval, about twice as long as wide, with a row of setae along lateral to terminal margin. Flagellum shorter than peduncle, composed of eight articles, each article with three pairs of thin setae and one long seta with aesthetascs.

***Antenna*** (Fig. 2B–D): Peduncle composed of three articles. Article 1 with one rounded process on anterior margin, covered by eight setae (Fig. 2C). Article 2 about twice as long as wide, with a stout spine at dorsodistal margin. Article 3 longer than article 2, with one plumose seta on proximal margin and several rows of spines or setae along medial anterior margin (Fig. 2D), as follows:

1. Proximal row of seven setae and robust plumose seta on inner surface.
2. Five spines and nine spine-like setae along proximal half, distalmost the longest.
3. Six spine-like setae on external lateral face.
4. Seven plumose setae, two thin plumose setae, and two simple setae, each associated with a row of proximal spines.
5. Eight robust spines increasing in length distally, one robust plumose seta, two simple setae, one long simple seta and one long plumose seta at apex, 16 plumose setae arising from posterior distal margin, and two robust plumose and three thin simple setae arising from posterior proximal margin.

Flagellum longer than peduncle, composed of 18 articles, each article with two pairs of robust setae and one thin seta on anterior margin, and a single thin seta on posterior margin of articles 1–14.

***Mandible*** (Fig. 1F): Well developed. Mandibular palp composed of three articles. Article 2 sub-equal in length to article 3, article 2 with one thin and one long plumose seta at mid-length on lateral margin and sub-terminally on superior margin, respectively. Article 3 cylindrical, with marginal row of setae covering distal four-fifths of article, terminal margin with a simple long, a plumose long seta and serration spines (Fig. 1G). Molar process shorter than palp article 1, distal margin with row of teeth forming grinding surface. Incisor process broad basally with acute terminal process and a minute tooth along lateral margin.

***First maxilla*** (Fig. 1H): Proximal endite (Fig. 1I) with rounded medial margin, bearing 11 long plumose and three short robust plumose setae. Distal endite (Fig. 1J) with two rows of stout setae, spatulate setae, and a long plumose seta. Palp long, ca. six times longer than both endites combined, bearing nine setae.

***Second maxilla*** (Fig. 1K): Protopod subdivided into four endites bearing plumose setae. Endite 1 approximately as long as endite 3; endite 2 oval, smaller than endites 1 and 3; endite 4 smaller than endites 1–3. Endopod composed of two articles; article 1 subequal to article 2, lateral margin with plumose setae, article 2 with three terminal plumose setae. Exopod just reaching distal end of endopod article 1, bearing 14 plumose setae on lateral margins and three on terminal margin.

***Thoracopod 1*** (Fig. 3A): Endopod composed of one large and four distal small articles, with numerous plumose setae along outer margin. Exopod oval, not surpassing distal article of endopod, with three long plumose setae on terminal margin and 20 thin plumose setae along inner margin. Epipod smaller than that of thoracopods 3–7, tip of proximal lobe reaching beyond basis, distal lobe short and not reaching distal half of endopod.

***Thoracopods 3–7*** (Fig. 3B–F): Similar in shape. One long and 18 thin plumose setae on thoracopod 3 exopod, one long and 12 thin in thoracopod 4, one long and 15 thin on thoracopod 5, one long and 11 thin on thoracopod 6, and ten thin on thoracopod 7. Three thin plumose setae on thoracopod 6 epipod, eight thin on thoracopod 7, thoracopods 3–5 lacking setae, and thoracopod 2 damaged.

***Thoracopod 8*** (Fig. 3G): Smaller than that of other thoracopods. Endopod composed of a large article and four small articles, with numerous plumose setae along outer margin. Exopod narrow with lateral margin concave, with two long plumose setae on terminal margin. Epipod large, distal lobe elongate, with 12 plumose setae.

***Pleon*** (Fig. 1A): Composed of seven segments. Posterior margin of pleonite 1 smooth, pleonite 2 with rounded and acute denticles on half-length of posterior margin with flat margin between acute denticles. Pleonite 3 with rounded denticles. Pleonite 4 with rounded denticles, posterolateral margin expanded forming a narrow acute process. Pleonites 5–7 with rounded denticles (Fig. 5C).

***Pleopod 1*** (Fig. 4A): Protopod twice as long as wide, with single seta on proximal lateral margin, three setae on medial inner margin, with a simple seta, a simple spine-like seta, and a plumose seta near base of endopod; and a spine-like seta and simple seta near base of exopod. Endopod composed of two segments, longer than exopod, distal segment with an acute process at apex, bearing a long robust simple spine, lateral and medial margins each with plumose setae, appendix interna of proximal segment with three short, recurved hooks (Fig. 4B). Exopod with five simple spines along lateral margin, three simple spines on distolateral margin, the distal one the longest, and plumose setae along inner margin.

***Pleopod 2*** (Fig. 4C): Protopod twice as long as wide, six simple setae on proximal face, a single spine-like seta on proximal inner margin, one thin seta and a long spine-like seta on proximal outer margin, four setae on medial outer margin, two short and three long setae near base of endopod, single spine-like seta and robust simple spine near base of exopod, seven simple setae on distal face, with blade-like process between exopod and endopod. Endopod composed of two segments, longer than exopod, distal segment with acute process at apex, bearing a long robust simple spine, lateral and medial margins each with plumose setae, appendix interna of proximal segment with three short, recurved hooks. Exopod with a row of five pairs of simple spines and a thin plumose seta along lateral margin and single thin plumose seta near basis, two simple spines on distal margin, and plumose setae along inner margin.

***Pleopod 3*** (Fig. 4D): Protopod twice as long as wide, with seven simple setae on proximal face, one simple spine-like seta on proximal inner margin, a single spine-like seta on proximal outer margin, three simple setae near base of endopod, two simple setae, a robust seta, and a simple spine-like seta near base of exopod, eight simple setae on distal face, with a blade-like process between the exopod and endopod. Endopod composed of two segments, longer than exopod, distal segment with an acute process at apex, bearing a long robust simple spine, lateral and medial margins each with plumose setae, appendix interna of proximal segment with three short, recurved hooks. Exopod with a row of six pairs of simple spines and thin plumose setae along lateral margin, two simple spines on distal margin, and plumose setae along inner margin.

***Pleopod 4*** (Fig. 4E): Protopod sub-rectangular, both terminal sides acute, twice as long as wide, with nine simple setae on proximal face, one spine-like seta and five simple setae on proximal inner margin, four simple setae on proximal lateral margin, and six simple setae on distal face. Endopod composed of two segments, longer than exopod, distal segment with an acute process at apex, bearing a long robust simple spine, lateral and medial margins each with plumose setae, appendix interna of proximal segment with three short, recurved hooks. Exopod with a row of seven pairs of simple spines and thin plumose setae along lateral margin, two stout simple spines on distal margin, and plumose setae along inner margin.

***Pleopod 5*** (Fig. 4F): Composed of two segments. Distal segment about three times as long as wide, bearing eight simple spines and 18 simple thin setae, lateral margin with approximately 13 simple setae.

***Pleopod 6*** (Fig. 4G): Rami bearing five simple spines and six simple thin setae, lateral margin with three simple thin and one simple long seta.

***Anal plate*** (Fig. 5B): No distinct ‘shoulder’, point acute. Furcal rami (Fig. 5A) shorter than pleonite 7 and telson combined, 24 spines along lateral margin, eight spine-like setae and 13 plumose setae along inner margin, and two robust setae on distolateral margin.

###### Morphological variation.

The number of distal setae of the antennule peduncle article 4 and of lateral spines of the pleopod 1 exopod show some variation among specimens: six to nine setae on distal margin, and six simple spines on lateral margin, respectively.

###### Etymology.

The name *agoensis* refers to the type locality of the new species, the sea near Ago Bay.

###### Remarks.

The seven known species of the genus *Sarsinebalia* can be grouped according to presence or absence of the ommatidia and the pigment of eyes. Three species, *S.biscayensis* Ledoyer, 1998, *S.typhlops* (Sars, 1869) and *S.pseudotyphlops* Petryashov, 2016, lack both ommatidia and pigments. On the contrary, *S.cristoboi* Moreira, Gestoso & Troncoso, 2003, *S.kunyensis* Ledoyer, 2000, *S.ledoyeri* Moreira, Esquete & Cunha, 2021, and *S.urgorrii* Moreira, Gestoso & Troncoso, 2003, have compound eyes provided with ommatidia; *S.cristoboi*, *S.kunyensis* and *S.urgorrii* also bear red eye pigment. Therefore, *S.agoensis* sp. nov. is close the latter four species for the presence of ommatidia, but differs from them in a number of features (Table 1):

**Table 1.** Comparison of *Sarsinebaliaagoensis* sp. nov. with related species of *Sarsinebalia*. Abbreviations: a = article; An2 = antenna; enp = endopod; exp = exopod; Mx2 = second maxilla; pn 6–7 = pleonite 6–7; pp1 = pleopod 1; sp = supraocular plate.

Species	Ommatidia	Eye pigment	Eye shape	Eye-stalk terminal lobes	Sp length	An2 a1 distal process	Mx2 exp	Pp1 exp lateral margin	Shape of pn 6–7 denticles	Source
*S.agoensis* sp. nov.	Present	Absent	Almost oblong, disto-ventral margin concave	3 lobes	Two thirds of eye-stalk	Covered by 8 setae	< enp a1	4 simple spines	Distally rounded	This paper
*S.kunyensis Ledoyer*, 2000	Present	Absent	Oval, elongated	Absent	Two thirds of eye-stalk	Margin smooth	> enp a1	7 serrated spines	Elongated, distally rounded	Ledoyer (2000)
*S.cristoboi* Moreira et al., 2003	Present	Present (red-orange)	Expanded distally	Absent	Beyond distal end of eye-stalk	One tooth (2?)	> enp a1	Smooth	Distally rounded	Moreira et al. (2003)
*S.urgorrii* Moreira et al., 2003	Present	Present (red-orange, dark)	Oblong	Absent	Two thirds of eye-stalk	One thooth	> enp a1	Smooth	Distally bluntly rounded	Moreira et al. (2003)
*S.ledoyeri* Moreira et al., 2021	Present	Absent	Almost oblong, disto-ventral margin concave	Absent	Half of eye-stalk	One tooth	> enp a1	Smooth	Elongated, slightly triangular distally	Moreira et al. (2021)

1. *S.agoensis* sp. nov. is the only known species in the genus with three lobes on the terminal margin of the eyestalk; in *S.cristoboi* the eyestalk is slightly longer than wide and the distal border is straight; in *S.kunyensis* it is oval and tapering distally, oblong in *S.urgorrii*, and oblong with concave disto-ventral margin in *S.ledoyeri*.
2. The tip of the supraocular plate extends along the proximal half of the eyestalk in *S.agoensis* sp. nov. and *S.ledoyeri*, along the proximal two-thirds in *S.kunyensis* and *S.urgorrii*, and beyond the terminal margin of the eyestalk in *S.cristoboi*.
3. Article 1 of the antenna peduncle has a rounded process covered with eight setae in *S.agoensis* sp. nov. This process ends in one tooth in *S.ledoyeri*, *S.cristoboi*, and *S.urgorrii*; apparently, there is a second tooth/spine in *S.cristoboi* that is not mentioned in the original description but illustrated (cfr. fig. 2A in Moreira et al. 2003). The process is smooth and lacking teeth/spines in *S.kunyensis*.
4. The tip of the exopod of the second maxilla just reaches the distal end of the first article of the endopod in *S.agoensis* sp. nov., but extends well beyond the level of the second article of the endopod in *S.cristoboi*, *S.kunyensis*, *S.ledoyeri*, and *S.urgorrii*.
5. The lateral margin of the pleopod 1 exopod bears a row of four to six simple spines in *S.agoensis* sp. nov., whereas *S.kunyensis* bears seven serrated spines; the lateral margin is smooth in *S.cristoboi*, *S.ledoyeri*, and *S.urgorrii*.
6. The denticles of the posterior margins of pleonites 6–7 are distally rounded in *S.agoensis* sp. nov. and *S.cristoboi*, elongated and distally rounded in *S.kunyensis*, distally bluntly rounded in *S.urgorrii*, and elongated and distally slightly triangular in *S.ledoyeri*.

In conclusion, *S.agoensis* sp. nov. stands out from other known species of *Sarsinebalia* by having the eyestalk provided with distal lobes and pleopod 1 exopod with a lateral row of several spines.

Until now, there have been few studies on Leptostraca in waters near Japan; in addition, only limited sea areas have been studied. For this reason, previous studies have never been able to accurately evaluate the diversity of Leptostraca in waters near Japan. Ago Bay is also one of the sea areas that has never been investigated, and this paper is the first report from this area. This is also the first report of the genus *Sarsinebalia* in waters near Japan. The discovery of *S.agoensis* sp. nov. suggests that further investigations may uncover further diversity of Leptostraca in waters near Japan.

### ﻿Key to species of the genus *Sarsinebalia*

**Table:** 

1	Eyestalk lacking ommatidia or pigment	**2**
–	Eyestalk provided with ommatidia or pigment	**4**
2	Eyestalk oblong	***S.pseudotyphlops* Petryashov, 2016**
–	Eyestalk not oblong	**3**
3	Eyestalk sub-rectangular; disto-ventral margin concave	***S.typhlops* (Sars, 1870)**
–	Eyestalk elongated; disto-ventral margin convex	***S.biscayensis* Ledoyer, 1998**
4	Eyestalk with three lobes on terminal margin	***S.agoensis* sp. nov.**
–	Eye-stalk lacking terminal lobes	**5**
5	Eyestalk expanding distally	***S.cristoboi* Moreira, Gestoso & Troncoso, 2003**
–	Eyestalk tapering distally	**6**
6	Eyestalk oval	***S.kunyensis* Ledoyer, 2000**
–	Eyestalk not oval	**7**
7	Eyestalk oblong	***S.urgorrii* Moreira, Gestoco & Troncoso, 2003**
–	Eyestalk almost oblong; disto-ventral margin concave	***S.ledoyeri* Moreira, Esquete & Cunha, 2021**

## Supplementary Material

XML Treatment for Sarsinebalia agoensis
